# Differential Proteomics in Dequeened Honeybee Colonies Reveals Lower Viral Load in Hemolymph of Fertile Worker Bees

**DOI:** 10.1371/journal.pone.0020043

**Published:** 2011-06-15

**Authors:** Dries Cardoen, Ulrich R. Ernst, Matthias Van Vaerenbergh, Bart Boerjan, Dirk C. de Graaf, Tom Wenseleers, Liliane Schoofs, Peter Verleyen

**Affiliations:** 1 Research Group of Functional Genomics and Proteomics, K.U.Leuven, Leuven, Belgium; 2 Laboratory of Entomology, K.U.Leuven, Leuven, Belgium; 3 Laboratory of Zoophysiology, Ghent University, Ghent, Belgium; Institut Européen de Chimie et Biologie, France

## Abstract

The eusocial societies of honeybees, where the queen is the only fertile female among tens of thousands sterile worker bees, have intrigued scientists for centuries. The proximate factors, which cause the inhibition of worker bee ovaries, remain largely unknown; as are the factors which cause the activation of worker ovaries upon the loss of queen and brood in the colony. In an attempt to reveal key players in the regulatory network, we made a proteomic comparison of hemolymph profiles of workers with completely activated ovaries vs. rudimentary ovaries. An unexpected finding of this study is the correlation between age matched worker sterility and the enrichment of *Picorna*-like virus proteins. Fertile workers, on the other hand, show the upregulation of potential components of the immune system. It remains to be investigated whether viral infections contribute to worker sterility directly or are the result of a weaker immune system of sterile workers.

## Introduction

Honeybees have fascinated scientists for hundreds of years because of the highly evolved eusocial organization of their colonies in which labor is divided on several levels. The queen monopolizes egg laying, while thousands of female workers perform all other tasks in the colony. This extreme form of worker altruism involves the sterility of the vast majority (99.9%) of the worker bee caste, with only very few exceptions (half a dozen bees in a colony of several then thousand workers) [1]. However, in a hopelessly queenless colony, i.e. when the queen dies and queen breeding fails, worker bees are able to activate their ovaries. The fertile workers produce viable, unfertilized, haploid eggs, which develop, as in most haplodiploid species, into males. Many theoretical predictions concerning the evolutionary background of this remarkable trait have been proposed [2], [3]. In brief, theory predicts honeybees are selected to police (i.e. remove) any worker laid egg because, on average, each bee is more closely related to the sons of the queen compared to sons of any other worker. Because policing is highly efficient (99% of worker laid eggs are removed [4]) this causes a social pressure in which all workers remain sterile, except for 0.1- 0.01% of cheaters [5]. The proximate mechanisms, however, which cause the inhibition of worker bee ovaries, remain partially unknown; as are the factors which cause the activation of worker ovaries upon the loss of queen and brood in the colony.

Honeybee communication relies partly on pheromone signaling, which is detected at the antennae. The binding of pheromones to their receptors triggers brain responses, which in turn provides a signal towards the rest of the body if needed. There is no neural connection towards the ovaries and thus the hemolymph, the insect equivalent of vertebrate blood which circulates in an open system, is likely to reflect the physiological state and hence differences between fertile and sterile workers. In addition, it has already been suggested that hemolymph factors might influence honeybee egg production [6] Therefore we compared the hemolymph proteome profiles of age matched fertile and sterile workers collected from the same hopelessly queenless colony by means of 2D-DIGE. It is already known that queen and brood pheromones influence worker bee physiology in such a way that, among others, worker bees stay sterile in the presence of those pheromones [7]–[10]. The inhibition of the sterility is lifted upon the absence of the pheromones (i.e. a hopelessly queenless condition) in approximately 30% of the workers in a colony [11]. Both queen and brood pheromone treatments of caged bees are known to cause the differential expression of large gene sets in an age dependent way. In young bees, genes associated with nursing behaviour are upregulated and genes associated with foraging behavior are downregulated. In old bees the opposite happens [12], [13]. However, the overlap of the differentially expressed gene sets is weak, suggesting independent physiological pathways. The regulatory networks for initiating and maintaining worker ovary activation have thoroughly been studied in the brain or very young bees by means of microarray analyses [14]–[16]. In honeybees, proteomic approaches have been used for, e. g. investigation of embryonic caste determination [17] and worker task specialization [18], but not in relation to fertility of workers.

Our major finding involves the differential detection of an RNA virus. This *Picorna*-like RNA virus is up to 20-fold enriched in the sterile workers. Reproductive workers on the other hand show the upregulation of several potential components of the immune system, such as serine proteases and a peroxidase, which could indicate an activated immune system. In case of Deformed wing virus, infections are mostly covert, i.e. infected bees have no distinct phenotype; only a low number of bees suffer from an overt infection and have deformed wings or no wings at all [19]. Hence, these findings should also prompt researchers to be aware of possible covert viral infections in colonies as they can influence any honeybee experiment.

## Materials and Methods

### Honeybees and sample collection

Honeybees were reared at the beekeeping facility of the Laboratory of Zoophysiology (Ghent, Belgium), according to standard beekeeping methods. Two colonies, headed by a singly mated queen (obtained from the DLR Fachzentrum für Bienen und Imkerei, Mayen, Germany), were dequeened on July 9^th^ 2009 to induce worker egg-laying. Starting the same day, the emerging brood was paint-marked on the thorax and reintroduced. To this end, brood frames were placed in an incubator (Memmert Precision Incubator Model INE 700, 34°C, high relative humidity) allowing the brood to emerge. Every 24 h, newly emerged bees were marked with a colored dot (paint markers, Posca and acryl paint, Lefranc & Bourgeois) on the thorax according to the day of emergence and were reintroduced into the original beehive. All queen breeding attempts were annihilated (both queen rearing cell and its larva were removed) and worker bees of 18 days old were collected each time in the early morning from the hopelessly queenless colonies, starting July 27^th^. Therefore, all marked bees of 18 days old were collected after opening the hive. Bees were anesthetized on ice prior to dissection. Hemolymph was collected according to Bogaerts *et al*. [20] and temporarily stored by placing the 1 µl glass capillaries on a stainless steel plate cooled (approximately −50°C) by means of dry ice, until ovary scoring by means of dissection. Ovaries containing at least one egg larger than 1.1 mm were scored as fully activated and will throughout this paper be referred to as ovaries of fertile workers. Those without visible oocytes or oocytes smaller than 0.3 mm were noted as completely inactivated ovaries and will be referred to as ovaries of sterile workers. Honeybees with intermediate ovary activation were not included in the 2D-DIGE analysis. After ovary scoring, the capillaries were thawed and the hemolymph was immediately transferred in approximately an equal volume of lysis solution (150 mM NaCl, 10 mM KCl, 4 mM CaCl_2_, 2 mM MgCl_2_, 10 mM 2-[4-(2-hydroxyethyl)piperazin-1-yl]ethanesulfonic acid and Protease inhibitor cocktail (Roche)). Sample pooling between different colonies was avoided in order to minimize within replicate variation due to genetic and colony factors. Thus only hemolymph from full sister workers bees was pooled.

### 2D Electrophoresis and DIGE

In total, 5 independent biological replicates from 2 colonies were analyzed. Fluorescent labeling was performed as described by Haenen *et al.* [21]. Preparation of analytical and preparative gels and gel electrophoresis was performed as described by Bogaerts *et al*. [20] and are described at http://miapegeldb.expasy.org/experiment/112/gel/253. Images were imported into Progenesis SameSpots (Nonlinear Dynamics) and the raw spot volumes were exported. The values were normalized within the gels (loess) and between gels (quantile). Finally, differential expression was measured according to the Log_2_ spot volume ratios and its statistical significance was assessed using empirical Bayes moderated t-tests carried out in *limma*. In this analysis, colony was included as a random blocking variable and *p*-values were corrected following the Benjamini-Hochberg procedure [22], to control the false discovery rate. Spot excision of the differentially detected spots by means of a spotpicker (GE Healthcare) from preparative gels and in gel tryptic digests were performed as described by Bogaerts *et al.* [23].

### Protein identification

**Figure 1:**
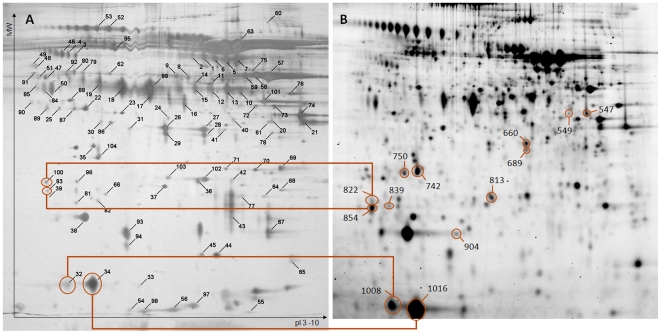
Display of the hemolymph proteome of honeybee workers (non linear pI range of 3–10, 24 cm). (A) The reference work by Bogaerts *et al.* [20] (Copyright Wiley-VCH GMbH & Co. KGaA. reproduced with permission) and (B) this experiment. All identified proteins are depicted and spot numbers correspond to Table 1. Spots identified by means of spot matching are indicated with the corresponding spot in the reference work.

**Figure 2:**
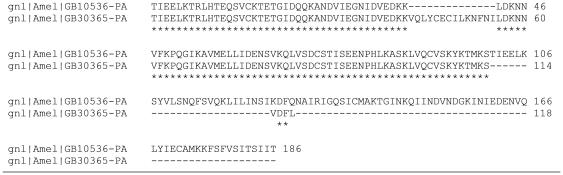
Alignment of GB10536 and GB30365 by means of ClustalW. Sequences are obtained from the honeybee official gene set prerelease 2 available at http://genomes.arc.georgetown.edu/Amel_pre_release2_OGS_pep.fa. Results suggest that both proteins are splice variants of the same protein, which might have been annotated as two distinct genes.

Tryptic digests were desalted by Ziptip C_18_ and 1.5 µl of the elution was loaded on a stainless steel target plate together with the same volume of a saturated solution of α-cyano-4-hydroxycinnamic acid matrix in 50% acetonetrile and 0.5% formic acid in Milli-Q water. The automated MALDI-TOFTOF (Ultraflex II, Bruker Daltonics) selected the 12 most intense peaks of the MS spectrum, which were analyzed in the LIFT mode. Spectra from one spot were combined, using BioTools (Bruker Daltonics) and analyzed using the MS/MS ion search tool from Mascot [24] against the in-house *Apis mellifera* database, based on the official gene set prerelease 2 (available at beebase.org) and the publically available NCBI nr database, covering all non redundant sequences available at NCBI. Virus proteins were identified by Mascot searches against the honeybee official gene set release 1, as they were removed in the prerelease 2. Details of identifications are summarized in Table S1. As spots 822, 854, 1008 and 1016 (Table 1) were faint on the preparative gels, we identified them by spot matching to a reference spot map of Bogaerts *et al.* who used exactly the same protocols and instruments [20] (Fig. 1). Spots 1008 and 1016 (Table 1) have both been identified as GB30365 and GB10536. After GO-term inspection (both odorant binding) and sequence alignment, we came to the conclusion that we are probably dealing with splice variants of the same protein which might have been annotated as two distinct genes (Fig. 2) and both spots will be referred to as protein GB10536.

**Table 1:** All identified differentially expressed proteins with spot number, fold change (FC = fertile/sterile), the *p*-value adjusted by the method of Benjamini-Hochberg, the method of identification (Mascot MSMS Ion searches or spot matching to the reference work of Bogaerts *et al.* [20]), the identification (GB numbers from the beebase official gene set prerelease 2, or release 1 for the viruses) and annotation of each protein are displayed.

Spot Nr	FC	Adj *p*-value	IDprocess	Identification	Annotation
750	−39.49	0.0072	MSMS Ion	GB19460-PA	similartoAldolase CG6058-PF
742	−20.36	0.0073	MSMS Ion	polyprotein[Deformed wing virus]	(RNA virus)
813	−20.30	0.0072	MSMS Ion	polyprotein[Deformed wing virus]	(RNA virus)
547	−19.02	0.0072	MSMS Ion	polyprotein[Kakugo virus]	(RNA virus)
549	−10.86	0.0072	MSMS Ion	polyprotein[Kakugo virus]	(RNA virus)
660	−3.60	0.0292	MSMS Ion	GB10708	hypothetical LOC408807
689	−3.43	0.0447	MSMS Ion	GB10708	hypothetical LOC408807
1008	4.29	0.0446	Spot match	GB10536	Odorant binding protein (obp) 14
822	5.06	0.0247	Spot match	GB10192	similar to CG5867-PA
536	5.11	0.0364	MSMS Ion	GB18767	Serine Protease 8
839	5.76	0.0179	MSMS Ion	GB11665	similar to Imaginal disc growth factor 4 CG1780-PA
854	5.80	0.0179	Spot match	GB11298	cSPH 42 similar to CG5390-PA
1016	6.48	0.0179	Spot match	GB10536	Odorant binding protein (obp) 14
904	8.30	0.0179	MSMS Ion	GB19380	similar to thioredoxin peroxidase 1 CG1633-PA

Additional information is displayed in Table S1.

## Results and Discussion

In total, only 18 out of 847 detected and matched spots were found to be differentially expressed, 9 of which were relatively upregulated in the hemolymph of sterile worker bees and 9 were more abundant in the fertile worker bees (Table 1). Protein identifications of spots upregulated in the sterile workers resulted in 7 identified spots. Four spots were surprisingly identified as the polyprotein of a RNA virus. Seven spots of the 9 upregulated in fertile workers were identified, resulting in 6 unique proteins (Table 1). No overlap with earlier microarray studies has been found [15], [16].

### Proteins upregulated in reproductive worker hemolymph

Three out of the six identified proteins upregulated in reproductive workers have previously been shown to be present in queen spermathecae [25]; i.e. GB11665, GB19380 and GB10536. We can rule out any contamination with spermatheca proteins as the hemolymph was collected at the wing joints to the thorax and thus not in the proximity of the, rudimentary, worker spermatheca in the abdomen. The gene coding for protein GB11665, which is annotated as an imaginal disc growth factor, but which has the GO-terms chitinase and glycosylase assigned, was previously identified from honeybee venom gland tissue [26] and was upregulated in worker larvae in a microarray study which compared developing queens and worker bees [27]. GB19380, annotated as thioredoxin peroxidase 1, has been categorized as a gene overexpressed in queen larvae [28]. Thioredoxin peroxidase 1 was also identified as a component of honeybee venom gland tissue [26]. In addition, peroxidases are known to be involved in the response to oxidative stress, which can be caused by viral infections [29], [30]. In *Bombyx mori* this enzyme, though absent from the hemolymph, was detected in fat bodies and the midgut and upregulated upon baculovirus infection [31]. Thus it is possible that the upregulation of this enzyme is closely linked to a differential response against the RNA-virus as discussed below. Thioredoxin peroxidase is also reported to be an apoptosis inhibitor [32]. The same protein is more abundant in activated ovaries compared to rudimentary ones (personal communication). Consequently, the hemolymph thioredoxin peroxidase might serve in the ovaries to prevent apoptosis and promote ovary growth and egg development. The third protein which previously has also been found expressed in spermathecae is the odorant binding protein 14 (OBP14, GB10536). OBPs are defined as small (12–18 kDa) soluble proteins, which are able to reversibly bind small hydrophobic molecules [33]. These carrier proteins serve as a shuttle for the small odorant molecules (e.g. pheromones) towards the odorant receptors [34]. They are important for pheromone signalling in honeybees [34] and other insect species [35], [36] and for the detection of plant odours [37]. *Obp*14 transcripts have been detected in developing larvae [38]; the protein itself in venom gland tissue [26] and hemolymph [20], but were absent in eggs and pupae [39]. However, the function of odorant binding proteins in body parts, others than the antennae, remains elusive [38].

In addition, Serine protease 8 (SP8, GB18767) and a Serine protease homolog (cSPH42, GB11298) are more abundant in fertile worker hemolymph. The occurrence of proteases in hemolymph samples may point to a contamination by a damaged digestive system. However, we are confident this is not the case because the sample collection involved no grave damage to the body in the proximity of the digestive tract and the bleeding was induced only by removing the wings and gently pressing the thorax. Previously, cSPH42 transcripts have been found to be differentially expressed in honeybee larvae and adults upon microbial infection [40]. This might point to an enhanced immune system in fertile workers or to an ongoing inflammatory response reaction. GB10192, which is 5-fold upregulated in fertile workers, has no reported functional or transcriptional information in honeybees yet. Moreover, little is known about the homologous gene in *Drosophila melanogaster*, CG5867, although InterproScan predicts a Juvenile Hormone binding function for this protein [20]. Juvenile Hormone (JH) is known to be involved in the regulation of oogenesis in numerous insect species [41], [42] and developing queen larvae show different JH titer patterns compared to worker larvae [43]–[45]. But in worker bees, JH has lost its oogenic function and has instead acquired a new function related to the worker division of labor [42], [46].

Particularly remarkable was the non differential status of the major yolk protein Vitellogenin (Vg). One would expect this particular protein to be strongly upregulated in the fertile worker bees, as Vg circulates in the hemolymph and is associated with egg production. Queens do have significantly higher circulating Vg titers and higher transcript abundances in fat bodies compared to workers [47], [48] and Vg levels seem to be an indicator for queen ovary development [49]. In worker bees, Vg either seems not to be an indicator of fertility at all, as was the case in another RNA study concerning the honeybee worker reproductive behaviour [14] or the circulating Vg-titers might only be temporarily elevated in fertile workers.

### Upregulated in sterile workers

GB19460, annotated as an Aldolase, is nearly 40-fold upregulated (*p* = 0.007) in sterile workers. Aldolases have an enzymatic function in glycolysis, necessary in providing energy from sugar substrates. This protein has previously been found to be upregulated in workers compared to queens [27] and nearly significantly upregulated (*p* = 0.055) in sterile workers compared to fertile workers in a whole body microarray screenings (personal communication). In the latter study, genes associated with, among others, an increased energy metabolism, flight and foraging behavior, positive phototaxis, flight and heart muscle contraction were upregulated in sterile workers, suggesting that these workers were foraging compared to fertile workers. Intriguingly, the homologous gene in *Drosophila*, is known to be involved in mesoderm development [50], and more specifically at the M band and Z disc of flying muscles [51]. The upregulation of Aldolase in the hemolymph of sterile workers might thus be related with higher muscle activity and foraging. Besides muscle tissue, this protein has also been detected in honeybee queen spermathecae [25] and drone seminal vesicles [52]. The presence of Aldolase in hemolymph of sterile workers seems contradictory to its presence in reproductive organs, but the latter is most likely explained by the general function of this protein which is the production of energy out of sugar. Sperm cells do not have mitochondria and rely completely on glycolysis for energy production. After copulation, they are kept alive for several years in the queen spermatheca. Finally, *Drosophila melanogaster* Aldolase (CG6058) was found to be significantly upregulated in hemolymph of infected larvae [53].

The most remarkable result of this study is the massive enrichment of polyproteins of two honeybee viruses, belonging to the *Iflaviridae*, the Kakugo virus (KV) and the Deformed wing virus (DWV) in sterile workers. The viruses were identified in 4 different spots, with ratios ranging from a 10-fold to a more than 20-fold change (*p*<0.01) and have previously been identified in the hemolymph of random bees caught at the entrance of a bee hive [20]. Our data strongly suggest a viral infection of most sterile workers, whereas the fertile worker bees of the same age and from the same colony had much lower viral loads. We already mentioned that the serine proteases and the thioredoxin peroxidase, which are probably players of the insect innate immune system [29], [30], [40], are upregulated in the reproductive workers, where relatively few virus particles have been found. This suggests that reproductive workers (partly) activated their immune systemwhich resulted in lower viral loads. One of the two proteins upregulated in the sterile, infected bees (GB10708) is strongly correlated with KV infection and is known to be expressed in peripheral regions of the brain [54]. This protein was found to be induced in the hemolymph upon microbial infection [55]. Inspection of the GO-terms, obtained via Blast2Go [56], suggests it to be either a phospholipase or an insulin-like growth factor binding protein. However, this protein identified from 2 different spots (ratio≈3.5, *p*<0.05), was assumed to be a Hymenopteran specific gene and little is known about the nature of the protein [54]. All information from our analysis points to the heavy infection of all sterile workers compared with the low viral loads of fertile worker bees, which seem to have beaten the viral infection quite successfully.

KV and DWV have initially been identified in different contexts, i.e. deformed wings of severely infected bees for DWV and aggressive behaviour for KV, yet their genome is highly similar [57]. Despite the distinct infection phenotypes, the polyproteins of KV, DWV and even another virus, Varroa desctructor virus (VDV) are extremely similar [19], [58], [59]. Based on the sequence data from the MALDI-TOF TOF analysis and subsequent Mascot searches against the honeybee official gene set release 1, spots 547 and 549 were identified as the polyprotein from KV and spot 742 and 813 as the DWV polyprotein. However, upon inspection of the sequences and alignments to the published protein sequences [58]–[60] the spots 547, 549 and 742 match the DWV and VDV and spot 813 matches KV and VDV (Fig. S1). No distinction between VDV and the other viruses can be made based on current sequence data. Note that RNA-viruses are susceptible to mutations [61], which makes the distinction between DWV, VDV and KV even more difficult, even with other techniques such as (real time) PCR. Possibly, the viruses mentioned here might all be variants of the same virus, as recently, more natural recombinants have been identified [58]. In addition, no bees with deformed wings were included in the sample collection; consequently we are dealing with a mild infection rate, even in the sterile workers. The 4 identified spots represent 3 proteins which are processed from the virus polyprotein. Spots 547 and 549 are probably Virus protein (VP) 1, spot 742 is VP2 and spot 813 is VP3. The three proteins are the main structural proteins [57] which encapsulate the viral RNA-genome. Other proteins, which are part of the polyprotein, such as a RNA helicase, RNA dependent RNA polymerase and chymotrypsin protease have not been identified in this study.

Very recently, viral infections have been associated with the degeneration of queen ovaries [62]. It is tempting to speculate that the virus infection induces or promotes the sterility in these queenless worker bees. However, we cannot rule out other hypotheses, for instance that the highly infected bees were in a poorer condition *ab initio* and therefore were neither able to activate their immune system sufficiently nor to activate their ovaries. Another explanation might be that the sterile workers effectively start to forage earlier, which is suggested to be correlated with a lower immune response capacity [63]; however this is still under debate [64], [65]. Future experiments with infected and non-infected bees, e.g. treated with RNAi targeting the viral RNA, will reveal the impact of viral RNA on ovary maturation.

From another point of view, it is intriguing that honeybee viruses, which are highly common in European and North American honeybee populations, are capable of deflecting the results of scientific experiments. Here, we aimed at identifying proteins relevant for ovary (in)activity, but ended up finding the proteome profiles of hemolymph more affected by either the presence or absence of virus particles and proteins related to immune defense. In other words, while investigating any aspect of honeybee physiology, an underlying infection (viruses in particular or pathogens in general) might largely affect the outcome and possibly account for contradictory results. Part of the problem is that infections are sometimes not easily detected. In the case of DWV, for instance, only bees with severe DWV-infection have the typical deformed wings or no wings at all and bees with mild infection rates are not to be distinguished from healthy bees. However, the detection of the viral proteins in the hemolymph might be the basis for a new protein based diagnostic test for the Deformed wing virus.

## Supporting Information

Table S1 **Protein identification data displaying spot number, identified protein, MSMS Ion search score and the number of sequenced peptides matched to the protein.** For each peptide the sequence, individual peptide score, the E-value and the searched database are displayed.
(XLS)

Figure S1 **Alignment of Kakugo virus (KV), Deformed wing virus (DWV) and Varroa destructor virus (VDV) (by ClustalW).** Aligned sequences are obtained from the honeybee official gene set release 1 available at http://genomes.arc.georgetown.edu/Amel_release1_OGS_pep.fa, which were the basis of the Mascot virus identifications (KV_mascot and DWV_mascot). In addition the most recent protein sequences of both KV (KV_Fujiyuki_2004), VDV (VDV_Ongus_2004) and DWV (DWV-Moore_2011) have been aligned in order to reveal the identity of the virus involved in this experiment. The highlighted parts are the sequences which are included in the Mascot identification, based on sequence data obtained from Maldi TOF TOF analysis. Based on current information, VP1 and VP2 are identical to DWV proteins but VP3 fits better with a KV protein. However, the DWV, KV and VDV are highly similar viruses and difficult to distinguish on the protein level. Probably they are variants of the same virus. The borders of VP1, VP2 and VP3 are indicated.
(PDF)
