# Supplementary material for: Differential Proteomics in Dequeened Honeybee Colonies Reveals Lower Viral Load in Hemolymph of Fertile Worker Bees
Source: PLoS One. 2011 Jun 15;6(6):e20043. doi: 10.1371/journal.pone.0020043 (PMC3115943; doi:10.1371/journal.pone.0020043)
Supplement: Figure S1 — Alignment of Kakugo virus (KV), Deformed wing virus (DWV) and Varroa destructor virus (VDV) (by ClustalW). Aligned sequences are obtained from the honeybee official gene set release 1 available at http://genomes.arc.georgetown.edu/Amel_release1_OGS_pep.fa, which were the basis of the Mascot virus identifications (KV_mascot and DWV_mascot). In addition the most recent protein sequences of both KV (KV_Fujiyuki_2004), VDV (VDV_Ongus_2004) and DWV (DWV-Moore_2011) have been aligned in order to reveal the identity of the virus involved in this experiment. The highlighted parts are the sequences which are included in the Mascot identification, based on sequence data obtained from Maldi TOF TOF analysis. Based on current information, VP1 and VP2 are identical to DWV proteins but VP3 fits better with a KV protein. However, the DWV, KV and VDV are highly similar viruses and difficult to distinguish on the protein level. Probably they are variants of the same virus. The borders of VP1, VP2 and VP3 are indicated. (PDF) [file pone.0020043.s002.pdf]

Supplemental File 1

Alignment of Kakugo virus (KV), Deformed wing virus (DWV) and Varroa destructor virus (VDV) (by ClustalW). Aligned sequences are obtained from the honeybee official gene set release 1 available at http://genomes.arc.georgetown.edu/Amel_release1_OGS_pep.fa, which were the basis of the Mascot virus identifications (KV_mascot and DWV_mascot). In addition the most recent protein sequences of both KV (KV_Fujiyuki_2004), VDV (VDV_Ongus_2004) and DWV (DWV-Moore_2011) have been aligned in order to reveal the identity of the virus involved in this experiment. The highlighted parts are the sequences which are included in the Mascot identification, based on sequence data obtained from Maldi TOF TOF analysis. Based on current information, VP1 and VP2 are identical to DWV proteins but VP3 fits better with a KV protein. However, the DWV, KV and VDV are highly similar viruses and difficult to distinguish on the protein level. Probably they are variants of the same virus. The borders of VP1, VP2 and VP3 are indicated.

Spot 547 (identified as Kakugo virus): VP1
Spot 549 (identified as Kakugo virus): VP1
Spot 742 (identified as Deformed wing virus): VP2
Spot 813 (identified as Deformed wing virus): VP3

```
KV_mascot                         MAFSCGTLSYAAVAQAPSVAHAPRSWEIDEARRRRVIKRLALEQERIRNV 50
VDV_Ongus_2004                    MAFSCGTLSYAAVAQAPSVAHAPRSWEIDEARRRRVIKRLALEQERIRNV 50
DWV_mascot                        MAFSCGTLSYAAVAQAPSVAHAPRSWEIDEARRRRVIKRLALEQERIRNV 50
DWV-Moore_2011                    MAFSCGTLSYAAVAQAPSVAHAPRSWEIDEARRRRVIKRLALEQERIRNV 50
KV_Fujiyuki_2004                  MAFSCGTLSYSAVTQAPSVAYAPRTWEVDEARRRRVIKRLALEQERIRNV 50
                                  **********:**:******:***:**:**********************

KV_mascot                         LDVTVYDHTTWEQEDARDNEFLMEQLNNLYTIYSIAERCTRRPVQEHVPI 100
VDV_Ongus_2004                    LDVTVYDHTTWEQEDARDNEFLMEQLNNLYTIYSIAERCTRRPVQEHVPI 100
DWV_mascot                        LDVTVYDHTTWEQEDARDNEFLTEQLNNLYTIYSIAERCTRRPVQEHVPI 100
DWV-Moore_2011                    LDVTVYDHTTWEQEDARDNEFLTEQLNNLYTIYSIAERCTRRPVQEHVPI 100
KV_Fujiyuki_2004                  LDVDVYNQATWEQEDVRDNEFLTEQLNNLYTIYSIAERCTRRPIKECSPI 100
                                  *** **:::******.****** ********************::*  **

KV_mascot                         SISNRYSPLESLKIEVGKDAGEFVFKKPKYTKICKKVKRVASKFVREKVV 150
VDV_Ongus_2004                    SISNRYSPLESLKIEVGKDAGEFVFKKPKYTKICKKVKRVASKFVREKVV 150
DWV_mascot                        SISNRYSPLESLKIEVGEDAGEFVFKKPKYTKICKKVKRVASKFVREKVV 150
DWV-Moore_2011                    SISNRYSPLESLKIEVGKDAGEFVFKKPKYTKICKKVKRVASKFVREKVV 150
KV_Fujiyuki_2004                  SVSNRFAPLESLKVEIGQEASECIFKKPKYTRVCKKVKRVATRFVREKVV 150
```

```
                                 *:***::******:*:*::*.* :*******::********::*******
KV_mascot                        RPVCNRSPMLLFKIKKVIYDLHLYRLRKQVRLLRREKQREYELECVTSLL 200
VDV_Ongus_2004                   RPVCNRSPMLLFKIKKVIYDLHLYRLRKQVRLLRREKQREYELECVTSLL 200
DWV_mascot                       RPVCNRSPMLLFKIKKVIYDLHLYRLRKQVRLLRREKQREYELECVTSLL 200
DWV-Moore_2011                   RPVCNRSPMLLFKIKKVIYDLHLYRLRKQVRLLRREKQREYELECVTSLL 200
KV_Fujiyuki_2004                 RPMCPRSPMLLFKLKKIIYDLHLYRLRKQIRMLRRQKQRDYELECVTNLL 200
                                 **:* ********:**:************:*:***:***:*******.**

KV_mascot                        QLSNPVSAKPEMDNPNPGPDGEGEVELEKDSNVVLTTQRDPSTSIPAPTS 250
VDV_Ongus_2004                   QLSNPVSAKPEMDNPNPGPDGEGEVELEKDSNVVLTTQRDPSTSIPAPTS 250
DWV_mascot                       QLSNPVSAKPEMDNPNPGPDGEGEVELEKDSNVVLTTQRDPSTSIPAPTS 250
DWV-Moore_2011                   QLSNPVSAKPEMDNPNPGPDGEGEVELEKDSNVVLTTQRDPSTSIPAPTS 250
KV_Fujiyuki_2004                 QLSNPVQAKPEMDNPNPGPDGEGEVELEKDSNVVLTTQRDPSTSIPAPVS 250
                                 ******.*****************************************.*
                                        |=> VP2

KV_mascot                        VKWSRWTSNDVVDDYATITSRWYQIAEFVWSKDDPFDKELARLILPRALL 300
VDV_Ongus_2004                   VKWSRWTSNDVVDDYATITSRWYQIAEFVWSKDDPFDKELARLILPRALL 300
DWV_mascot                       VKWSRWTSNDVVDDYATITSRWYQIAEFVWSKDDPFDKELARLILPRALL 300
DWV-Moore_2011                   VKWSRWTSNDVVDDYATITSRWYQIAEFVWSKDDPFDKELARLILPRALL 300
KV_Fujiyuki_2004                 VKWSRWTSNDVVDDYATITSRWYQIAEFVWSKDDPFDKELARLILPRALL 300
                                 **************************************************

KV_mascot                        SSIEANSDAICDVPNTIPFKVHAYWRGDMEVRVQINSNKFQVGQLQATWY 350
VDV_Ongus_2004                   SSIEANSDAICDVPNTIPFKVHAYWRGDMEVRVQINSNKFQVGQLQATWY 350
DWV_mascot                       SSIEANSDAICDVPNTIPFKVHAYWRGDMEVRVQINSNKFQVGQLQATWY 350
DWV-Moore_2011                   SSIEANSDAICDVPNTIPFKVHAYWRGDMEVRVQINSNKFQVGQLQATWY 350
KV_Fujiyuki_2004                 SSIEANSDAICDVPNTIPFKVPAYWRGDMEVRVQISSNKFQVGQLQATWY 350
                                 ********************* *************.**************

KV_mascot                        YSDHENLNIQTKRSVYGFSHMDHALISASASNEAKLVIPFKHVYPFLPTR 400
VDV_Ongus_2004                   YSDHENLNIQTKRSVYGFSHMDHALISASASNEAKLVIPFKHVYPFLPTR 400
DWV_mascot                       YSDHENLNIQTKRSVYGFSHMDHALISASASNEAKLVIPFKHVYPFLPTR 400
DWV-Moore_2011                   YSDHENLNIQTKRSVYGFSHMDHALISASASNEAKLMIPFKHVYPFLPTR 400
KV_Fujiyuki_2004                 YSDHENLNISSKRSVYGFSQMDHALISASASNEAKLVIPFKHVYPFLPTR 400
                                 *********.:********:****************:*************
```

```
KV_mascot          VVPDWTTGILDMGTLNIRVIAPLRMSATGPTTCNVVVFIKLNNSEFTGTS 450
VDV_Ongus_2004     VVPDWTTGILDMGTLNIRVIAPLRMSATGPTTCNVVVFIKLNNSEFTGTS 450
DWV_mascot         VVPDWTTGILDMGTLNIRVIAPLRMSATGPTTCNVVVFIKLNNSEFTGTS 450
DWV-Moore_2011     VVPDWTTGILDMGTLNIRVIAPLRMSATGPTTCNVVVFIKLNNSEFTGTS 450
KV_Fujiyuki_2004   IVPDWTTGILDMGALNIRVIAPLRMSATGPTTCNVVVFIKLNNSEFTGTS 450
                   :************:************************************

KV_mascot          SGKFYANQIRAKPEMDRVLNLAEGLLNNTVGGCNMDNPSYQQSPRHFVPT 500
VDV_Ongus_2004     SGKFYANQIRAKPEMDRVLNLAEGLLNNTVGGCNMDNPSYQQSPRHFVPT 500
DWV_mascot         SGKFYANQIRAKPEMDRVLNLAEGLLNNTVGGCNMDNPSYQQSPRHFVPT 500
DWV-Moore_2011     SGKFYANQIRAKPEMDRVLNLAEGLLNNTVGGCNMDNPSYQQSPRHFVPT 500
KV_Fujiyuki_2004   SGKLYASQIRAKPEXDRILNLAEGLLNNTIGGNNMDNPSYQQSPRHFVPT 500
                   ***:**.******* **:***********:** *****************
                               VP2 <=|               |=> VP1

KV_mascot          GMHSLALGTNLVEPLHALRLDASGTTQHPVGCAPDEDMTVSSIASRYGLI 550
VDV_Ongus_2004     GMHSLALGTNLVEPLHALRLDASGTTQHPVGCAPDEDMTVSSIASRYGLI 550
DWV_mascot         GMHSLALGTNLVEPLHALRLDASGTTQHPVGCAPDEDMTVSSIASRYGLI 550
DWV-Moore_2011     GMHSLALGTNLVEPLHALRLDASGTTQHPVGCAPDEDMTVSSIASRYGLI 550
KV_Fujiyuki_2004   GMHSLALGTNLVEPLHALRLDAAGTTQHPVGCAPDEDMTVSSIASRYGLI 550
                   **********************:***************************

KV_mascot          RQVQWKKDHAKGSLLLQLDADPFVEQKIEGTNPISLYWFAPVGVVSSMFM 600
VDV_Ongus_2004     RQVQWKKDHAKGSLLLQLDADPFVEQKIEGTNPISLYWFAPVGVVSSMFM 600
DWV_mascot         RQVQWKKDHAKGSLLLQLDADPFVEQKIEGTNPISLYWFAPVGVVSSMFM 600
DWV-Moore_2011     RQVQWKKDHAKGSLLLQLDADPFVEQKIEGTNPISLYWFAPVGVVSSMFM 600
KV_Fujiyuki_2004   RQIQWKKDHAKGSLLLQLDADPFVEQRIEGTNPISLYWFAPVGVVSSMFM 600
                   **:***********************:***********************

KV_mascot          QWRGSLEYRFDIIASQFHTGRLIVGYVPGLTASLQRQMDYMKLKSSSYVV 650
VDV_Ongus_2004     QWRGSLEYRFDIIASQFHTGRLIVGYVPGLTASLQRQMDYMKLKSSSYVV 650
DWV_mascot         QWRGSLEYRFDIIASQFHTGRLIVGYVPGLTASLQRQMDYMKLKSSSYVV 650
DWV-Moore_2011     QWRGSLEYRFDIIASQFHTGRLIVGYVPGLTASLQRQMDYMKLKSSSYVV 650
KV_Fujiyuki_2004   QWRGSLEYRFDIIASQFHTGRLIVGYVPGLTASLQQQMDYMKLKSSSYVV 650
                   ***********************************:**************
```

```
KV_mascot            FDLQESNSFTFEVPYVSYRPWWVRKYGGNYLPSSTDAPSTLFMYVQVPLI 700
VDV_Ongus_2004       FDLQESNSFTFEVPYVSYRPWWVRKYGGNYLPSSTDAPSTLFMYVQVPLI 700
DWV_mascot           FDLQESNSFTFEVPYVSYRPWWVRKYGGNYLPSSTDAPSTLFMYVQVPLI 700
DWV-Moore_2011       FDLQESNSFTFEVPYVSYRPWWVRKYGGNYLPSSTDAPSTLFMYVQVPLI 700
KV_Fujiyuki_2004     FDLQESNSFTFEVPYVSYRPWWVRKYGGNYLPSSTDAPSTLFMYVQVPLI 700
                     **************************************************

KV_mascot            PMEAVSDTIDINVYVRGGSSFEVCVPVQPSLGLNWNTDFILRNDEEYRAK 750
VDV_Ongus_2004       PMEAVSDTIDINVYVRGGSSFEVCVPVQPSLGLNWNTDFILRNDEEYRAK 750
DWV_mascot           PMEAVSDTIDINVYVRGGSSFEVCVPVQPSLGLNWNTDFILRNDEEYRAK 750
DWV-Moore_2011       PMEAVSDTIDINVYVRGGSSFEVCVPVQPSLGLNWNTDFILRNDEEYRAK 750
KV_Fujiyuki_2004     PMEAVSDTIDINVYVRGGSSFEVCVPVQPSLGLNWNTDFILRNDEEYRAK 750
                     **************************************************

KV_mascot            NGYAPYYAGVWHSFNNSNSLVFRWGSASDQIAQWPTITVPRGELAFLRIR 800
VDV_Ongus_2004       NGYAPYYAGVWHSFNNSNSLVFRWGSASDQIAQWPTITVPRGELAFLRIR 800
DWV_mascot           NGYAPYYAGVWHSFNNSNSLVFRWGSASDQIAQWPTITVPRGELAFLRIR 800
DWV-Moore_2011       NGYAPYYAGVWHSFNNSNSLVFRWGSASDQIAQWPTITVPRGELAFLRIR 800
KV_Fujiyuki_2004     TGYAPYYAGVWHSFNNSNSLVFRWGSASDQIAQWPTISVPRGELAFLRIX 800
                     .************************************:***********

KV_mascot            DAKQAAVGTQPWRTMVVWPSGHGYNIGIPTYNAERARQLAQHLYGGGSLT 850
VDV_Ongus_2004       DAKQAAVGTQPWRTMVVWPSGHGYNIGIPTYNAERARQLAQHLYGGGSLT 850
DWV_mascot           DAKQAAVGTQPWRTMVVWPSGHGYNIGIPTYNAERARQLAQHLYGGGSLT 850
DWV-Moore_2011       DAKQAAVGTQPWRTMVVWPSGHGYNIGIPTYNAERARQLAQHLYGGGSLT 850
KV_Fujiyuki_2004     DGKXAAVGTQPWRTMVVWPSGHGYNIGIPTYNAERARQLAQHLYGGGSLT 850
                     *.* **********************************************

KV_mascot            DEKAKQLFVPANQQGPGKVSNGNPVWEVMRAPLATQQAHIQDFEFVEAVP 900
VDV_Ongus_2004       DEKAKQLFVPANQQGPGKVSNGNPVWEVMRAPLATQQAHIQDFEFVEAVP 900
DWV_mascot           DEKAKQLFVPANQQGPGKVSNGNPVWEVMRAPLATQQAHIQDFEFVEAVP 900
DWV-Moore_2011       DEKAKQLFVPANQQGPGKVSNGNPVWEVMRAPLATQQAHIQDFEFVEAVP 900
KV_Fujiyuki_2004     DEKAKQLFVPANQQGPGTVSNGNPVWEVMRAPLATQRAHVQDFEFIEAIP 900
                     *****************.******************:**:*****:**:*
```

```
KV_mascot          EGEESRNTTVLDTTTTLQSSGFGRAFFGEAFNDLKTLMRRYQLYGQLLLS 950
VDV_Ongus_2004     EGEESRNTTVLDTTTTLQSSGFGRAFFGEAFNDLKTLMRRYQLYGQLLLS 950
DWV_mascot         EGEESRNTTVLDTTTTLQSSGFGRAFFGEAFNDLKTLMRRYQLYGQLLLS 950
DWV-Moore_2011     EGEESRNTTVLDTTTTLQSSGFGRAFFGEAFNDLKTLMRRYQLYGQLLLS 950
KV_Fujiyuki_2004   EGEESRNTTVLDTTTTLQSSGFGRAFFGEAFNDLKTLMRRYQLYGQLLLS 950
                   ************** ***********************************
                  VP1 <=|=> VP3

KV_mascot          VTTDKDIDHCMFTFPCLPQGLALDIGSAGSPHEIFNRCRDGIIPLIASGY 1000
VDV_Ongus_2004     VTTDKDIDHCMFTFPCLPQGLALDIGSAGSPHEIFNRCRDGIIPLIASGY 1000
DWV_mascot         VTTDKDIDHCMFTFPCLPQGLALDIGSAGSPHEIFNRCRDGIIPLIASGY 1000
DWV-Moore_2011     VTTDKDIDHCMFTFPCLPQGLALDIGSAGSPHEIFNRCRDGIIPLIASGY 1000
KV_Fujiyuki_2004   VTTDKDIDHCMFTFPCLPQGLALDIGSAGSPHEIFNRCRDGIIPLIASGY 1000
                   **************************************************

KV_mascot          RFYRGDLRFKIVFPSNVNSNIWVQHRPDRRLKGWSEAKIVNCDAVSTGQG 1050
VDV_Ongus_2004     RFYRGDLRFKIVFPSNVNSNIWVQHRPDRRLKGWSEAKIVNCDAVSTGQG 1050
DWV_mascot         RFYRGDLRFKIVFPSNVNSNIWVQHRPDRRLKGWSEAKIVNCDAVSTGQG 1050
DWV-Moore_2011     RFYRGDLRFKIVFPSNVNSNIWVQHRPDRRLKGWSEAKIVNCDAVSTGQG 1050
KV_Fujiyuki_2004   RFYRGDLRYKIVFPSNVNSNIWVQHRPDRRLEGWSAAKIVNCDAVSTGQG 1050
                   ********:**********************:*** **************

KV_mascot          VYNHGYASHIQITRVNNVIELEVPFYNATCYNYLQAFNPSSAASSYAVSL 1100
VDV_Ongus_2004     VYNHGYASHIQITRVNNVIELEVPFYNATCYNYLQAFNPSSAASSYAVSL 1100
DWV_mascot         VYNHGYASHIQITRVNNVIELEVPFYNATCYNYLQAFNPSSAASSYAVSL 1100
DWV-Moore_2011     VYNHGYASHIQITRVNNVIELEVPFYNATCYNYLQAFNPSSAASSYAVSL 1100
KV_Fujiyuki_2004   VYNHGYASHIQITRVNNVIELEVPFYNATCYNYLQAFNASSAASSYAVSL 1100
                   **************************************.***********

KV_mascot          GEISVGFQATSDDIAAIVNKPVTIYYSIGDGMQFSQWVGYQPMMILDQLP 1150
VDV_Ongus_2004     GEISVGFQATSDDIAAIVNKPVTIYYSIGDGMQFSQWVGYQPMMILDQLP 1150
DWV_mascot         GEISVGFQATSDDIAAIVNKPVTIYYSIGDGMQFSQWVGYQPMMILDQLP 1150
DWV-Moore_2011     GEISVGFQATSDDIAAIVNKPVTIYYSIGDGMQFSQWVGYQPMMILDQLP 1150
KV_Fujiyuki_2004   GEISVGFQATSDDIASIVNKPVTIYYSIGDGMQFSQWVGYQPMMILDQLP 1150
                   ***************:**********************************
```

```
KV_mascot                 APVVRAVPEGPIAKIKNFFHQTADEVREAQAAKMREDMGIVVQDVIGELS 1200
VDV_Ongus_2004            APVVRAVPEGPIAKIKNFFHQTADEVREAQAAKMREDMGIVVQDVIGELS 1200
DWV_mascot                APVVRAVPEGPIAKIKNFFHQTADEVREAQAAKMREDMGIVVQDVIGELS 1200
DWV-Moore_2011            APVVRAVPEGPIAKIKNFFHQTADEVREAQAAKMREDMGIVVQDVIGELS 1200
KV_Fujiyuki_2004          APVVRAVPEGPIAKIKNFFHQTADEVREAQAAKMREDMGMVVQDVIGELS 1200
                          ***************************************:**********
                             VP3 <=|

KV_mascot                 QAIPDLQQPEVQANVFSLVSQLVHAIIGTSLKTVAWAIVSIFVTLGLIGR 1250
VDV_Ongus_2004            QAIPDLQQPEVQANVFSLVSQLVHAIIGTSLKTVAWAIVSIFVTLGLIGR 1250
DWV_mascot                QAIPDLQQPEVQANVFSLVSQLVHAIIGTSLKTVAWAIVSIFVTLGLIGR 1250
DWV-Moore_2011            QAIPDLQQPEVQANVFSLVSQLVHAIIGTSLKTVAWAIVSIFVTLGLIGR 1250
KV_Fujiyuki_2004          QAIPDLQQPEVQANVFSLVSQLVHAIIGTSLKTVAWAIVSIFVTLGLIGR 1250
                          **************************************************

KV_mascot                 EMMHSVITVVKRLLEKYHLATQPQESANSGTVISAIPEAPNAEAEEASAW 1300
VDV_Ongus_2004            EMMHSVITVVKRLLEKYHLATQPQESANSGTVISAIPEAPNAEAEEASAW 1300
DWV_mascot                EMMHSVITVVKRLLEKYHLATQPQESANSGTVISAVPEAPNAEAEEASAW 1300
DWV-Moore_2011            EMMHSVITVVKRLLEKYHLATQPQESANSGTVISAVPEAPNAEAEEASAW 1300
KV_Fujiyuki_2004          EMMHSVITVVKRLLEKYHLATQPQESASSSTVISAVPEAPNAEAEEASAW 1300
                          ***************************.*.*****:**************

KV_mascot                 VSIIYNGVCNMLNVAAQKPKQFKDWVKLATVDFSNNCRGSNQVFVFFKNT 1350
VDV_Ongus_2004            VSIIYNGVCNMLNVAAQKPKQFKDWVKLATVDFSNNCRGSNQVFVFFKNT 1350
DWV_mascot                VSIIYNGVCNMLNVAAQKPKQFKDWVKLATVDFSNNCRGSNQVFVFFKNT 1350
DWV-Moore_2011            VSIIYNGVCNMLNVAAQKPKQFKDWVKLATVDFSNNCRGSNQVFVFFKNT 1350
KV_Fujiyuki_2004          VSIIYNGVCNMLNVAAQKPKQFKDWVKLATVDFSNNCRGSNQVFVFFKNT 1350
                          **************************************************

KV_mascot                 FEVLKKMWGYVFCQSNPAARLLKAVNDEPEILKAWVKECLYLDDPKFRMR 1400
VDV_Ongus_2004            FEVLKKMWGYVFCQSNPAARLLKAVNDEPEILKAWVKECLYLDDPKFRMR 1400
DWV_mascot                FEVLKKMWGYVFCQSNPAARLLKAVNDEPEILKAWVKECLYLDDPKFRMR 1400
DWV-Moore_2011            FEVLKKMWGYVFCQSNPAARLLKAVNDEPEILKAWVKECLYLDDPKFRMR 1400
KV_Fujiyuki_2004          FEVLKKMWGYVFCQSNPAARLLKAVNDEPEILKAWVKECLYLDDPKFRMR 1400
                          **************************************************
```

```
KV_mascot                 RAHDQEYIERVFAAHSYGQILLHDLTAEMNQSRNLSVFTRVYDQISKLKT 1450
VDV_Ongus_2004            RAHDQEYIERVFAAHSYGQILLHDLTAEMNQSRNLSVFTRVYDQISKLKT 1450
DWV_mascot                RAHDQEYIERVFAAHSYGQILLHDLTAEMNQSRNLSVFTRVYDQISKLKT 1450
DWV-Moore_2011            RAHDQEYIERVFAAHSYGQILLHDLTAEMNQSRNLSVFTRVYDQISKLKT 1450
KV_Fujiyuki_2004          RAHDQEYIERVFAAHSYGQILLHDLTAEMNQSRNLSVFTRVYDQISKLKT 1450
                          **************************************************

KV_mascot                 DLMEMGSNPYIRRECFTICMCGASGIGKSYLTDSLCSELLRASRTPVTTG 1500
VDV_Ongus_2004            DLMEMGSNPYIRRECFTICMCGASGIGKSYLTDSLCSELLRASRTPVTTG 1500
DWV_mascot                DLMEMGSNPYIRRECFTICMCGASGIGKSYLTDSLCSELLRASRTPVTTG 1500
DWV-Moore_2011            DLMEMGSNPYIRRECFTICMCGASGIGKSYLTDSLCSELLRASRTPVTTG 1500
KV_Fujiyuki_2004          DLMEMGSNPYIRRECFTICMCGASGIGKSYLTDSLCSELLRASRTPVTTG 1500
                          **************************************************

KV_mascot                 IKCVVNPLSDYWDQCDFQPVLCVDDMWSVETSTTLDKQLNMLFQVHSPIV 1550
VDV_Ongus_2004            IKCVVNPLSDYWDQCDFQPVLCVDDMWSVETSTTLDKQLNMLFQVHSPIV 1550
DWV_mascot                IKCVVNPLSDYWDQCDFQPVLCVDDMWSVETSTTLDKQLNMLFQVHSPIV 1550
DWV-Moore_2011            IKCVVNPLSDYWDQCDFQPVLCVDDMWSVETSTTLDKQLNMLFQVHSPIV 1550
KV_Fujiyuki_2004          IKCVVNPLSDYWDQCDFQPVLCVDDMWSVETSTTLDKQLNMLFQVHSPIV 1550
                          **************************************************

KV_mascot                 LSPPKADLEGKKMRYNPEIFIYNTNKPFPRFDRIAMEAIYRRRNVLIECK 1600
VDV_Ongus_2004            LSPPKADLEGKKMRYNPEIFIYNTNKPFPRFDRIAMEAIYRRRNVLIECK 1600
DWV_mascot                LSPPKADLEGKKMRYNPEIFIYNTNKPFPRFDRIAMEAIYRRRNVLIECK 1600
DWV-Moore_2011            LSPPKADLEGKKMRYNPEIFIYNTNKPFPRFDRIAMEAIYRRRNVLIECK 1600
KV_Fujiyuki_2004          LSPPKADLEGKKMRYNPEIFIYNTNKPFPRFDRIAMEAIYRRRNVLIECK 1600
                          **************************************************

KV_mascot                 ANEEKKRGCKHCENNIPIAECSPKILKDFHHIKFRYAHDVCNSETTWSEW 1650
VDV_Ongus_2004            ANEEKKRGCKHCENNIPIAECSPKILKDFHHIKFRYAHDVCNSETTWSEW 1650
DWV_mascot                ASEEKKRGCKHCEDDIPIAECSPKMLKDFHHIKFRYAHDVCNSETTWSEW 1650
DWV-Moore_2011            ASEEKKRGCKHCENDIPIAECSPKMLQDFHHIKFRYAHDVCNSETTWSEW 1650
KV_Fujiyuki_2004          ASEEKKRGCKHCENDIPIAECSPKMLKDFHHIKFRYAHDVCNSETTWSEW 1650
                          *.***********::*********:*:***********************

KV_mascot                 MSYNEFLEWITPVYMANRRKANESFKMRVDEMQMLRMDEPLEGDNILNKY 1700
VDV_Ongus_2004            MSYNEFLEWITPVYMANRRKANESFKMRVDEMQMLRMDEPLEGDNILNKY 1700
DWV_mascot                MTYNEFLEWITPVYMANRRKANESFKMRVDEMQMLRMDEPLEGDNILNKY 1700
DWV-Moore_2011            MTYSEFLEWITPVYMANRRKANESFKMRVDEMQMLRMDEPLEGDNILNKY 1700
KV_Fujiyuki_2004          MTYNEFLEWITPVYMANRRKANESFKMRVDEMQMLRMDEPLEGDNILNKY 1700
```

```
                                   *:*.**********************************************
KV_mascot                          VEVNQRLVEEMKAFKERTLWADLQRVGSEISTSVKKALPTISITEKLPHW 1750
VDV_Ongus_2004                     VEVNQRLVEEMKAFKERTLWADLQRVGSEISTSVKKALPTISITEKLPHW 1750
DWV_mascot                         VEVNQRLVEEMKAFKERTLWSDLHRVGAEISASVKKALPTISITEKLPHW 1750
DWV-Moore_2011                     VEVNQRLVEEMKAFKERTLWSDLHRVGAEISASVKKALPTISITEKLPHW 1750
KV_Fujiyuki_2004                   VEVNQRLVEEMKAFKERTLWSDLHRVGAEISASVKKALPTISITEKLPHW 1750
                                   ********************:**:***:***:******************

KV_mascot                          TIQCGIAKPEMDHAYEVMSSYAAGMNAEIEAHEQVRRSSLECQYIEPSTS 1800
VDV_Ongus_2004                     TIQCGIAKPEMDHAYEVMSSYAAGMNAEIEAHEQVRRSSLECQYIEPSTS 1800
DWV_mascot                         TVQCGIAKPEMDHAYEVMSSYAAGMNAEIEAHEQVRRSSVEC-------- 1792
DWV-Moore_2011                     TVQCGIAKPEMDHAYEVMSSYAAGMNAEIEAHEQVRRSSVECQYAEPQAS 1800
KV_Fujiyuki_2004                   TVQCGIAKPEMDHAYEVMSSYAAGMNAEIEAHEQVRRSSVECQYAEPQAP 1800
                                   *:*************************************:**

KV_mascot                          RPLDEEGPTIDEELLGEVEFTSSALERLVDEGYITGKQKKYMATWCTKRR 1850
VDV_Ongus_2004                     RPLDEEGPTIDEELLGEVEFTSSALERLVDEGYITGKQKKYMATWCTKRR 1850
DWV_mascot                         --------------------------------------------------
DWV-Moore_2011                     RNPDDEGPTIDEELMGDTEFTSQALERLVDEGYITGKQKKYIATWCSKRR 1850
KV_Fujiyuki_2004                   RNPDDEGPTIDEELMGDTEFTSQALERLVDEGYITGKQKKYIATWCSKRR 1850


KV_mascot                          EHVSDFDLVWTDNLRVLSAYVHERSTSTRLSTDDVKLFKTISMLHQRYDT 1900
VDV_Ongus_2004                     EHVSDFDLVWTDNLRVLSAYVHERSTSTRLSTDDVKLFKTISMLHQRYDT 1900
DWV_mascot                         --------------------------------------------------
DWV-Moore_2011                     EHTADFDLVWTDNLRVLSAYAHERSSSTRLSTDDVKLYKTISMLHQKYDT 1900
KV_Fujiyuki_2004                   EHTADFDLVWTDNLRVLSAYVHERSASTRLSTDDVKLYKTISMLHQKYDT 1900


KV_mascot                          TDCAKCQHWYAPLTAIYVDDRKLFWCQKETKTLIDVRKLSKEDVTVQSKL 1950
VDV_Ongus_2004                     TDCAKCQHWYAPLTAIYVDDRKLFWCQKETKTLIDVRKLSKEDVTVQSKL 1950
DWV_mascot                         --------------------------------------------------
DWV-Moore_2011                     TECAKCQHWYAPLTDIYVDDKKLFWCQKEKKTLIDVRKLSKEDVTVQSKL 1950
KV_Fujiyuki_2004                   TECAKCQHWYAPLTDIYVDDKKLFWCQKEKKTLIDVRKLSKEDVTVQSKL 1950


KV_mascot                          INLSVPCGDVCMLHSKYFNYLFHKAWLFENPTWRLIYNGTKKGMPEYFMN 2000
VDV_Ongus_2004                     INLSVPCGDVCMLHSKYFNYLFHKAWLFENPTWRLIYNGTKKGMPEYFMN 2000
DWV_mascot                         --------------------------------------------------
DWV-Moore_2011                     INLSVPCGEVCMLHSKYFNYLFHKAWLFENPTWRLIYNGTKKGMPEYFMN 2000
KV_Fujiyuki_2004                   INLSVPCGEVCMLHSKYFNYLFHKAWLFENPTWRLIYNGTKKGMPEYFMN 2000
```

```
KV_mascot                  CVDEISLDSKFCKVKVWLQAIIDKYLTRPVKMIRDFLFKWWPQVAYVLSL 2050
VDV_Ongus_2004             CVDEISLDSKFCKVKVWLQAIIDKYLTRPVKMIRDFLFKWWPQVAYVLSL 2050
DWV_mascot                 --------------------------------------------------
DWV-Moore_2011             CVDEISLDSKFGKVKVWLQAIIDKYLTRPVKMIRDFLFKWWPQVAYVLSL 2050
KV_Fujiyuki_2004           CVDEISLDSKFGKVKVWLQAIIDKYLTRPVKMIRDFLFKWWPQVAYVLSL 2050

KV_mascot                  LGIIGITAYEMRNPKSTAEDLAEHYVNRHCSSDFWSPGMATPQGLKYSEA 2100
VDV_Ongus_2004             LGIIGITAYEMRNPKSTAEDLAEHYVNRHCSSDFWSPGMATPQGLKYSEA 2100
DWV_mascot                 --------------------------------------------------
DWV-Moore_2011             LGIIGITAYEMRNPKPTSEELADHYVNRHCSSDFWSPGLASPQGLKYSEA 2100
KV_Fujiyuki_2004           LGIIGITAYEMRNPKPTSEQLADHYVNRHCSSDFWSPGLASPQGLKYSEA 2100

KV_mascot                  ITAKAPRIHRLPVTTRPQGSTQQVDAAVNKILQNMVYIGVVFPKVPGSKW 2150
VDV_Ongus_2004             ITAKAPRIHRLPVTTRPQGSTQQVDAAVNKILQNMVYIGVVFPKVPGSKW 2150
DWV_mascot                 --------------------------------------------------
DWV-Moore_2011             VTAKAPRIHRLPVTTKPQGSTQQVDAAVNKILQNMVYIGVVFPKVPGSKW 2150
KV_Fujiyuki_2004           VTAKAPRIHRLPVTTKPQGSTQQVDAAVNKILQNMVYIGVVFPKVPGSKW 2150

KV_mascot                  RDINFRCLMLHNRQCLMLRHYIESTAAFPEGTKYYFKYIHNQETRMSGDI 2200
VDV_Ongus_2004             RDINFRCLMLHNRQCLMLRHYIESTAAFPEGTKYYFKYIHNQETRMSGDI 2200
DWV_mascot                 --------------------------------------------------
DWV-Moore_2011             RDINFRCLMLHNRQCLMLRHYIESTAAFPEGTKYYFKYIHNQETRMSGDI 2200
KV_Fujiyuki_2004           RDINFRCLMLHNRQCLMLRHYIESTAAFPEGTKYYFKYIHNQETRMSGDI 2200

KV_mascot                  SGIEIDLLSLPRLYYGGLAGEESFDSNIVLVTMPNRIPECKSIVKFIASH 2250
VDV_Ongus_2004             SGIEIDLLSLPRLYYGGLAGEESFDSNIVLVTMPNRIPECKSIVKFIASH 2250
DWV_mascot                 --------------------------------------------------
DWV-Moore_2011             SGIEIDLLNLPRLYYGGLAGEESFDSNIVLVTMPNRIPECKSIIKFIASH 2250
KV_Fujiyuki_2004           SGIEIDLLNLPRLYYGGLAGEESFDSNIVLVTMPNRIPECKSIIKFIASH 2250

KV_mascot                  AEHARAQNDGVLVTGEHTQLLAFENNNKTPISINADGLYEVILQGVYTYP 2300
VDV_Ongus_2004             AEHARAQNDGVLVTGEHTQLLAFENNNKTPISINADGLYEVILQGVYTYP 2300
DWV_mascot                 --------------------------------------------------
DWV-Moore_2011             NEHIRAQNDGVLVTGDHTQLLAFENNNKTPISINADGLYEVILQGVYTYP 2300
KV_Fujiyuki_2004           NEHIRAQNDGVLVTGDHTQLLAFENNNKTPISINADGLYEVILQGVYTYP 2300
```

```
KV_mascot                       YHGDGVCGSILLSRNLQRPIIGIHVAGTEGLHGFGVAEPLVHEMFTGKAI 2350
VDV_Ongus_2004                  YHGDGVCGSILLSRNLQRPIIGIHVAGTEGLHGFGVAEPLVHEMFTGKAI 2350
DWV_mascot                      --------------------------------------------------
DWV-Moore_2011                  YHGDGVCGSILLSRNLQRPIIGIHVAGTEGLHGFGVAEPLVHEMFTGKAI 2350
KV_Fujiyuki_2004                YHGDGVCGSILLSRNLQRPIIGIHVAGTEGLHGFGVAEPLVHEMFTGKAI 2350

KV_mascot                       ESEREPYDRVYELPLRELDESDIGLDTDLYPIGRVDAKLAHAQSPSTGIK 2400
VDV_Ongus_2004                  ESEREPYDRVYELPLRELDESDIGLDTDLYPIGRVDAKLAHAQSPSTGIK 2400
DWV_mascot                      --------------------------------------------------
DWV-Moore_2011                  ESEREPYDRVYELPLRELDESDIGLDTDLYPIGRVDAKLAHAQSPSTGIK 2400
KV_Fujiyuki_2004                ESEREPYDRVYELPLRELDESDIGLDTDLYPIGRVDAKLAHAQSPSTGIK 2400

KV_mascot                       KTLIHGTFDVWTEPNPMSSRDPRIAPHDPLKLGCEKHGMPCSPFNRKHLE 2450
VDV_Ongus_2004                  KTLIHGTFDVWTEPNPMSSRDPRIAPHDPLKLGCEKHGMPCSPFNRKHLE 2450
DWV_mascot                      --------------------------------------------------
DWV-Moore_2011                  KTLIHGTFDVRTEPNPMSSRDPRIAPHDPLKLGCEKHGMPCSPFNRKHLE 2450
KV_Fujiyuki_2004                KTLIHGTFDVRTEPNPMSSRDPRXAPHDPLKLGCEKHGMPCSPFNRKHLE 2450

KV_mascot                       LATTHLKEKLISVVKPINGCKIRSLQDAVCGVPGLDGFDSISWNTSAGFP 2500
VDV_Ongus_2004                  LATTHLKEKLISVVKPINGCKIRSLQDAVCGVPGLDGFDSISWNTSAGFP 2500
DWV_mascot                      --------------------------------------------------
DWV-Moore_2011                  LATNHLKEKLVSVVKPINGCKIRSLQDAVCGVPGLDGFDSISWNTSAGFP 2500
KV_Fujiyuki_2004                LATNHLKEKLVSVVKPINGCKIRSLQDAVCGVPGLDGFDSISWNTSAGFP 2500

KV_mascot                       LSSLKPPGSSGKRWLFDIELQDSGCYLLRGMRPELEIQLTTTQLMRKKGM 2550
VDV_Ongus_2004                  LSSLKPPGSSGKRWLFDIELQDSGCYLLRGMRPELEIQLTTTQLMRKKGM 2550
DWV_mascot                      --------------------------------------------------
DWV-Moore_2011                  LSSLKPPGTSGKRWLFDIELQDSGCYLLRGMRPELEIQLSTTQLMRKKGI 2550
KV_Fujiyuki_2004                LSSLKPPGASGKRWLFDIELQDSGCYLLRGMRPELEIQLSTTQLMRKKGI 2550

KV_mascot                       KPHTIFTDCLKDTCLPVEKCRIPGKTRIFSISPVQFTIPFRQYYLDFMAS 2600
VDV_Ongus_2004                  KPHTIFTDCLKDTCLPVEKCRIPGKTRIFSISPVQFTIPFRQYYLDFMAS 2600
DWV_mascot                      --------------------------------------------------
DWV-Moore_2011                  KPHTIFTDCLKDTCLPVEKCRIPGKTRIFSISPVQFTIPFRQYYLDFMAS 2600
KV_Fujiyuki_2004                KPHTIFTDCLKDTCLPVEKCRIPGKTRIFSISPVQFTIPFRQYYLDFMAS 2600
```

```
KV_mascot              YRAARLNAEHGIGIDVNSLEWTNLATSLSKYGTHIVTGDYKNFGPGLDSD 2650
VDV_Ongus_2004         YRAARLNAEHGIGIDVNSLEWTNLATSLSKYGTHIVTGDYKNFGPGLDSD 2650
DWV_mascot             --------------------------------------------------
DWV-Moore_2011         YRAARLNAEHGIGIDVNSLEWTNLATRLSKYGTHIVTGDYKNFGPGLDSD 2650
KV_Fujiyuki_2004       YRAARLNAEHGIGIDVNSLEWTNLATSLSKYGTHIVTGDYKNFGPGLDSD 2650

KV_mascot              VAASAFEIIIDWVLNYTEEDDKDEMKRVMWTMAQEILAPSHLCRDLVYRV 2700
VDV_Ongus_2004         VAASAFEIIIDWVLNYTEEDDKDEMKRVMWTMAQEILAPSHLCRDLVYRV 2700
DWV_mascot             --------------------------------------------------
DWV-Moore_2011         VAASAFEIIIDWVLHYTEEDNKDEMKRVMWTMAQEILAPSHLCRDLVYRV 2700
KV_Fujiyuki_2004       VAASAFEIIIDWVLHYTEEDNKDEMKRVMWTMAQEILAPSHLCRDLVYRV 2700

KV_mascot              PCGIPSGSPITDILNTISNCLLIRLAWQGITDLPLSEFSRHVVLVCYGDD 2750
VDV_Ongus_2004         PCGIPSGSPITDILNTISNCLLIRLAWQGITDLPLSEFSRHVVLVCYGDD 2750
DWV_mascot             --------------------------------------------------
DWV-Moore_2011         PCGIPSGSPITDILNTISNCLLIRLAWLGITDLPLSEFSQNVVLVCYGDD 2750
KV_Fujiyuki_2004       PCGIPSGSPITDILNTISNCLLIRLAWLGITDLPLSEFSQNVVLVCYGDD 2750

KV_mascot              LIMNVSDEMIDKFNAVTIGDFFSRYKMEFTDQDKSGNTVRWRTLQTATFL 2800
VDV_Ongus_2004         LIMNVSDEMIDKFNAVTIGDFFSRYKMEFTDQDKSGNTVRWRTLQTATFL 2800
DWV_mascot             --------------------------------------------------
DWV-Moore_2011         LIMNVSDNMIDKFNAVTIGKFFSQYKMEFTDQDKSGNTVKWRTLQTATFL 2800
KV_Fujiyuki_2004       LIMNVSDNMIDKFNAVTIGKFFSQYEMVFTDQDKSGNTVKWRTLQTATFL 2800

KV_mascot              KHGFLKHPTRPVFLANLDKVSIEGTTNWTHARGLGRRVATIENAKQALEL 2850
VDV_Ongus_2004         KHGFLKHPTRPVFLANLDKVSIEGTTNWTHARGLGRRVATIENAKQALEL 2850
DWV_mascot             --------------------------------------------------
DWV-Moore_2011         KHGFLKHPTRPVFLANLDKVSVEGTTNWTHARGLGRRTATIENAKQALEL 2850
KV_Fujiyuki_2004       KHGFLKHPTRPVFLANLDKVSVEGTTNWTHARGLGRRAATIENAKQALEL 2850

KV_mascot              AFGWGPEYFNHVRNTIKMAFDKLGIYEDLITWEEMDVRCYASA 2893
VDV_Ongus_2004         AFGWGPEYFNHVRNTIKMAFDKLGIYEDLITWEEMDVRCYASA 2893
DWV_mascot             -------------------------------------------
DWV-Moore_2011         AFGWGPEYFNYVRNTIKMAFDKLGIYEDLITWEEMDVRCYASA 2893
KV_Fujiyuki_2004       AFGWGPEYFNYVRNTIKMAFDKLGIYEDLITWEEMDVRCYASA 2893
```
